# Use of Haematological Changes as a Predictor of Dengue Infection among Suspected Cases at Kairuki Hospital in Dar Es Salaam, Tanzania: A Retrospective Cross Sectional Study

**DOI:** 10.24248/eahrj.v5i1.655

**Published:** 2021-06-11

**Authors:** Florence Salvatory Kalabamu, Shaaban Maliki

**Affiliations:** a Faculty of Medicine, Hubert Kairuki Memorial University; b Kairuki Hospital, Tanzania

## Abstract

**Background:**

Dengue is a viral disease transmitted by female Aedes mosquitoes which are commonly found in tropical and subtropical areas. There is a dramatic increase in annual incidence rate of dengue attributed to urbanisation, poor environmental management as well as increased people mobility. Outbreak of dengue have been reported in Tanzania in recent years with Dar es salaam being the most affected region. Dengue is associated with haematological derangements and itindicates the severity of the disease. These changes have not been well elucidated in Tanzanian patients. The aim of this study was to determine these derangements among dengue patients admitted at Kairuki hospital in Dar es salaam, and compare these changes with non-dengue febrile patients.

**Methods:**

A retrospective cross sectional study was conducted among patients who were suspected to have dengue; tested for dengue IgM and their Complete Blood Count were tested during the index illness. This information was obtained from Kairuki hospital laboratory database. Haematological parameters were compared between dengue and non-dengue patients using SPSS Version 20.0. Binary logistic regression analysis was used to determine haematological predictors of dengue positive results.

**Results:**

A total of 255 patients were enrolled, whereby 188(73.7%) were dengue positive and 67 (26.3%) were negative. Dengue patients had relatively low mean total white blood cell counts compared to non-dengue patients (Students test= −2.7; *p value=.007).* Furthermore, Mean lymphocyte count was significantly low in dengue patients compared to non-dengue patients (Student's (t) test=−5.1; *p<.001*). Other haematological parameters were not significantly different. Lymphopenia was a significant predictor for dengue positive results (Adjusted Odd Ration =5.26 (95% CI=2.28–12.2; *P value <.001*).

**Conclusion:**

Patients with dengue had significantly low total white blood cell and lymphocyte count compared to non-dengue febrile patients. Lymphopenia is a significant haematological predictor for dengue positive results. Case defining signs and symptoms combined with these haematological changes may be used by clinicians as a guide to order confirmatory test for suspected dengue cases.

## BACKGROUND

Dengue is a viral disease caused by the dengue virus that belongs into the *flaviviridae* family. There are 4 distinct dengue serotypes (DEN-1, DEN-2, DEN-3, and DEN-4). Despite their serotypical differences, they lead to the same clinical presentation. The infection by one serotype confers lifelong immunity to that particular serotype, but subsequent infection by other serotypes is associated with a more severe disease.^1^ Dengueis transmitted mainly by female *Aedes aegypti* and *Aedes albopictus* mosquitoes, which are mainly distributed in tropical and subtropical regions.^2^ There is a prevailing spread and increased cases of dengue fever. In 2017, it was estimated that 105 million people got infected with dengue, with 41,000 deaths, and an estimated incidence rate of 1,371 per 100,000 population.^3^ Variations in temperature, travel, rainfall, and degree of urbanisation are major influencers of transmission.^4^ Severe dengue is among the leading infectious cause of morbidity in Latin America and some Asian countries.^5^

Clinical presentation of dengue is not specific, thus, cannot be differentiated from other febrile illnesses on clinical grounds. In 2009, World Health Organization (WHO) developed clinical classification of dengue according to clinical signs and symptoms: dengue without warning signs, dengue with warning signs (abdominal pain, persistent vomiting, body fluid accumulation, mucosal bleeding, lethargy, hepatomegaly, elevated haematocrit, and rapid decrease in platelets count), and severe dengue.^6^ Severe dengue comprises of severe plasma leakage, severe bleeding and severe organ involvement.^6^ Most dengue infections are asymptomatic or mild disease. Only 10% develop severe disease. Case fatality rate of untreated or poorly treated severe cases is as high as 20%. However, with appropriate management and treatment, the mortality can be reduced up to 2%-5%.^8^ Tanzania has been experiencing sporadic dengue outbreaks. The most recent outbreaks occurred in 2010 followed by other outbreaks in 2012, 2013, 2014, 2018, and 2019. These occurred during the heavy rainy seasons that start from March through June. In the 2019 outbreak, the regions that were most affected included; Dar es salaam, Tanga, Pwani, Morogoro, Singida, and Kilimanjaro. In this episode, more than 3,500 cases and 3 deaths were reported^9^. Seroprevalence is also remarkable in other regions within Tanzania which signifies the wider spread of dengue in the country.^10,11,12,13^

Dengue is associated with several haematological changes such as leucopoenia, lymphopenia or lymphocytosis, thrombocytopenia and elevated haematocrit.^14,15,16,17,18,19^ These changes are immune mediated and may vary from one region or ethnic group to another due to heterogeneity and previous disease exposure.^20,21,22^ There is paucity of data describing these derangements in Tanzanian patients.

The aim of this study was to determine the haematological changes among patients with confirmed dengue infection compared to non-dengue patients with similar signs and symptoms attending Kairuki hospital in Dar es Salaam-Tanzania. If detected early, these changes may guide clinicians to perform specific confirmatory tests and provide appropriate management and counselling.

## MATERIALS AND METHODS

### Study Design and Study Site

We conducted a retrospective case-control study at Kairuki hospital which is a National referral level facility located in Kinondoni Municipal in Dar es salaam. It is also a teaching hospital for Hubert Kairuki Memorial University, providing both inpatient and outpatient care with capacity of up to 200 beds and an average of 600 outpatients per day.

### Study Population and Participants Selection

The study involved patients who visited Kairuki hospital in the period between April to June 2019, were suspected to be having dengue and subjected to dengue serology test. Criteria for inclusion in the study were; availability of basic demographic information (age, sex, and residence), availability of dengue test results and Complete Blood Count (CBC). Those with indeterminate dengue test results and missing or invisible values in CBC were excluded from the study. We used the hospital database and laboratory logbooks to get basic demographic information, dengue test results as well as haematological findings from the Complete Blood Count (Beckman-Coulter, Model Act 10, Brea, CA, USA,). Dengue test was regarded as positive if patients tested positive either for non-structural protein-1 antigen (NS1-antigen), or immunoglobin M (IgM) for dengue by SD Bioline Duo Rapid Test (Standard Diagnostic, Inc., Gyeonggi-do, Korea) or both.

### Sample Size Estimation

The minimum sample size was calculated using the formula for comparing 2 means from independent samples.^23^ The power of the study was set at 80% with type I(α) and II(β) error of 0.05 and 0.2 respectively. We used the total white blood cells as a reference value and assumed the Normal Standard Deviation of total white blood cells to be the same in both groups (dengue and non-dengue participants) at 1.5 × 10^9^/l. We calculated the sample size that would enable us to detect the difference (variance) in mean total white blood cell count between the groups (dengue and non-dengue participants) from 1 × 10^9^/L and above. The minimum sample size estimated in each group was calculated using the following formula:


$$n={\left(Z_{\alpha }+Z_{\beta }\right)}^{2}*2*\sigma^{2}/d^{2},$$

where:

n= minimum sample size in each group

Z_α_= Critical value at α= 0.05 which is 1.96

Z_β_=Critical value at β=0.2 which is 0.84

σ= standard deviation of the mean in both groups

*d* is the variance of the mean between the groups

Therefore,


$$n={\left(1.96+0.84\right)}^{2}*2*{\left(1.5*10^{9}\right)}^{2}/{\left(1*10^{9}\right)}^{2}=35.$$

Thus, the minimum sample size in each group was estimated to be 35 participants.

### Data Analysis

Data was analysed using Statistical Package for Social Sciences (SPSS) version 20.0 (IBM Corp., Armonk, NY, USA). Proportions and categorical variables were compared using Chi square, Spearman's or Kendall's Tau Test whenever appropriate with their respective odd ratios and 95% confidence intervals. Continuous variables (absolute counts) between groups (dengue and non-dengue patients) were compared using Student's (t) test. Binary logistic regression analysis was used to determine demographic and haematological predictors of dengue positive results. Probability (p-value) of less or equal to 0.05 was considered statistically significant.

### Ethical Considerations

Ethical clearance was obtained from the Ethical Review Committee of the Hubert Kairuki Memorial University teaching hospital, with approval number KH/RE/HKM/10/19. Strict confidentiality was maintained throughout data collection and analysis process. No personal identifiers were included in data mining and data analysis was performed anonymously.

## RESULTS

**FIGURE 1.**
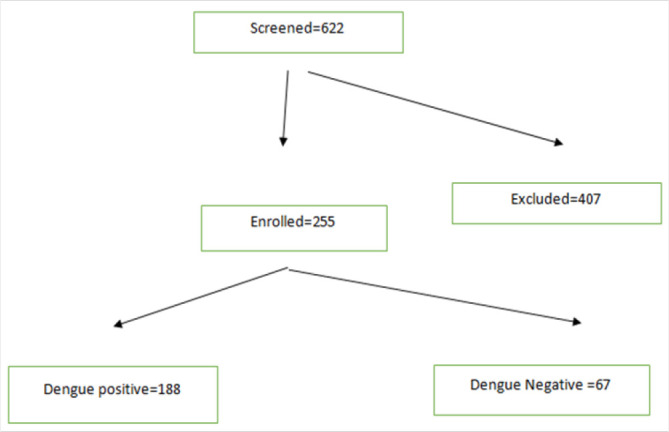
Screening and Enrolment Algorithm of Study Participants

A total of 255 participants were enrolled out of 662 patients who were subjected to dengue test. Participants who did not meet the selection criteria were excluded. Those were patients who had inadequate information, or patients who did not have complete blood count results (figure 1). Among the enrolled, 188(73.7%) tested positive for dengue while 67 (26.3%) tested negative. 215(84.3%) were between 41 and 60 year of age, while 8(3.5%) were below 20 years of age (Table 1)

**TABLE 1:** Baseline Characteristics of Study Participants (N=255)

Variable	Dengue Test Results			Chi-Square (X2)	*p-Value*
	Positive N(%)	Negative N(%)	Total N(%)		
**Age of participants**					
Below 20 years	8 (4.3)	1 (1.5)	9 (3.5)	1.46	.7
Between 21–40 years	11 (5.9)	3 (4.5)	14 (5.5)		
Between 41–60 years	156 (83.0)	59 (88.1)	215 (83.3)		
Above 60 years	13 (6.9)	4 (6)	17 (6.7)		
**Total**	118 (100)	67 (100)	255		
**Sex**					
Male	100 (53.2)	30 (44.8)	130 (51)	1.4	.24
Female	88 (46.8)	37 (55.2)	125 (49)		
**Total**	188 (100)	67 (100)	255 (100)		
**Attendance (Month)**					
April	70 (37.2)	38 (56.7)	108 (42.4)	7.7	.006***
May	118 (62.8)	29 (43.3)	147 (57.6)		
**Total**	188 (100)	67 (100)	255 (100)		
**Residence (Municipal)**					
Kinondoni	84 (44.7)	25 (37.3)	109 (42.7)	4.6	.31
Ubungo	34 (18.1)	12 (17.9)	46 (18)		
Ilala	24 (12.8)	12 (17.9)	36 (14.1)		
Temeke	18 (9.6)	3 (3)	21 (8.2)		
Kigamboni	28 (14.9)	15 (22.4)	43 (16.9)		
**Total**	188 (100)	67 (100)	255 (100)		

**FIGURE 2.**
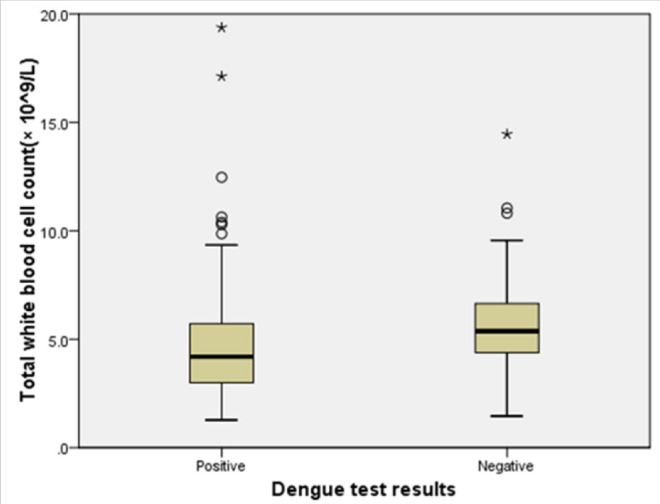
Difference in Mean Total White Blood Cell Count between Dengue and Non-Dengue Febrile Patients at Kairuki Hospital, 2019

Most of the participants (42.7%) were residing in Kinondoni Municipal. Sexes were equally distributed among dengue positive and negative patients. Those with positive dengue results had relatively low mean total white blood cell counts compared do dengue negative participants [Student's test= −2.7; *p value=.007* (figure 2)].

Mean total white blood cell count for dengue positive=4.7×10^9^/L. Mean Total white blood cell count for dengue negative=5.6×10^9^/L (Students (t) test= −2.7; *p=.007*)

**FIGURE 3.**
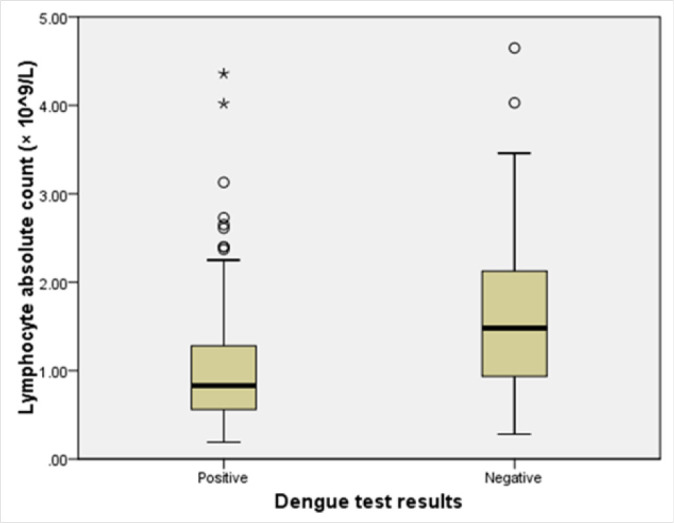
Difference in Mean Lymphocyte Count between Dengue and Non-Dengue Febrile Patients at Kairuki Hospital, 2019

In addition, mean lymphocyte count was significantly low in dengue patients compared to non-dengue patients [Students (t) test=−5.1; *p<.001* (figure 3)].

Mean lymphocyte for dengue positive=1.01×10^9^/L. Mean lymphocyte count for dengue negative=1.61×10^9^/L Students (t) test=−5.1; *p>.001*

**FIGURE 4.**
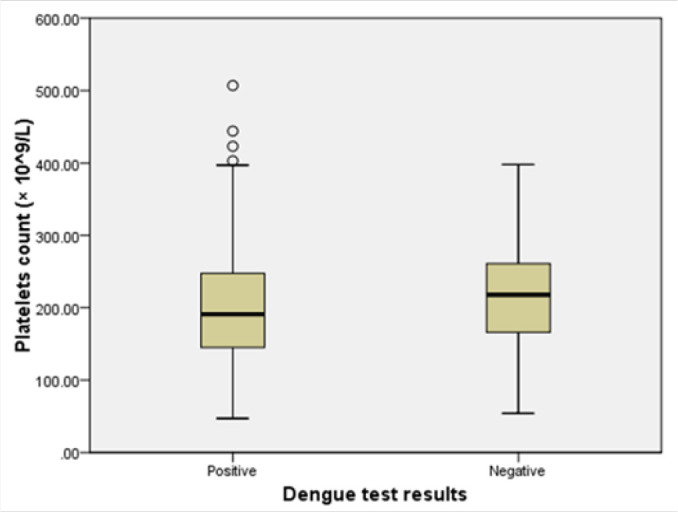
Difference in Mean Platelet Count between Dengue and Non-Dengue Febrile Patients At Kairuki Hospital, 2019

Patients with dengue had low mean platelet count compared to non-dengue patients, but the difference was not statistically significant [Student's test (t)=−0.86; *p=.39* (figure 4)].

Mean platelets count for dengue positive patients=202.7×10^9^/Mean platelets count for dengue negative patients=2 12.9×10^9^/L (Student's test (t)=−0.86; p=0.39); *p=.39*)

After controlling the confounding variables by stratification of total white blood count, lymphocyte count, and platelets, lymphocyte count was found to be highly associated with dengue positive results (Adjusted Odd Ratio = 5.26 (95% CI=2.28–12.2; *P value <.001*) (table 2).

**TABLE 2:** Social Demographic and Haematological Predictors of Dengue Positive Results among Febrile Patients At Kairuki Hospital, 2019

Variables	Dengue serology test			COR	95% Cl		*P* value	AOD	95% Cl		*P* value
	Positive N (%)	Negative N (%)	TOTAL		Lower	Upper			Lower	Upper	
**Residence(Municipal)**											
Kinondoni	84(37.3)	25(44.7)	109(42.7) Ref								
Ubungo	34(18.1)	12(17.9)	46(18.0)	1.800	0.834	3.887	0.135				
Ilala	24(12.8)	12(17.9)	36(14.1)	1.518	0.612	3.76	0.368				
Temeke	18(9.6)	3(4.5)	21(8.2)	1.071	0.421	2.728	0.885				
Others	28(14.9)	15(22.4)	43(16.9)	3.214	0.814	12.6	0.096	2.991	0.640	13.97	0.164
TOTAL	188(100)	67(100)	225(100)								
**Age of participants**											
Below 20 years	8(4.3)	1(1.5)	9(3.5) Ref								
Between 21–40 years	11(4.9)	3(4.5)	14(5.5)	2.462	0.232	26.1	0.455	3.727	0.267	51.93	0.328
Between 41–60 years	59(88.1)	156(83)	215(84.5)	1.128	0.206	6.16	0.889				
Above 60 years	13(6.9)	4(6.0)	17(6.7)	0.814	0.255	2.59	0.727				
TOTAL	188(100)	67(100)	255(100)								
**Sex**											
Male	100(53.2)	30(44.8)	130(52)	1.402	0.800	2.45	0.238	1.334	.665	2.675	0.418
Female	88(46.8)	37(55.2)	125(49) Ref								
TOTAL	188(100)	67(100)	255(100)								
**Platelet count(×l0^9^/L)**											
0.Normal (100–300)	144(76.6)	52(77.6)	196(76.9) Ref								
Low (<100)	19(10.1)	7(10.4)	26(10.2)	0.886	0.376	2. OS	0.782				
Elevated>300	25(13.3)	8(11.9)	33(12.9)	0.869	0.268	2.SI	0.814				
TOTAL	188(100)	67(100)	225(100)								
**Hematocrit (%)**											
Low (<35)	52(27.7)	17(25.4)	69(27.1) Ref								
Normal (35–55)	134(71.3)	48(71.6)	182(71.4)	3.059	0.400	23.4	0.282	7.256	.287	183.7	0.229
Elevated (>55)	2(1.1)	2(3)	4(1.6)	2.792	0.383	20.3	0.311	1.785	.099	32.26	0.695
TOTAL	188(100)	67(100)	255(100)								
**Haemoglobin(g/dl)**											
Low (<12)	45(23.9)2	0(29.9)	65(25.50 Ref								
Normal (12–16)	140(74.5)	45(67.2)	185(72.5)	1.500	0.232	9.6	0.670				
Elevated (>16)	3(1.6)	2(3)	5(2)	2.074	0.336	12.8	0.432				
TOTAL	188(100)	67(100)	255(100)								
**Red blood cell count (×l0^12^/L)**											
Low (<3.5)	6(3.2)	2(3)	8(3.1) Ref								
Normal (3.5–5.5)	170(90.4)	58(86.6)	228(89.4)	1.750	.275	11.1	0.554				
Elevated (>5.5)	12(6.4)	7(10.4)	19(7.5)	1.710	.643	4.5	0.283				
TOTAL	188(100)	67(100)	255(100)								
**Oesonophil absolute count (× 10^9^/L)**											
Low (<0.02)	5(2.7)	1(1.5)	6(2.4) Ref								
Normal (0.02–0.5)	182(96.8)	63(94)	245(96.1)	15.	0.663	339.5	0.089	9.508	.233	388.4	0.234
Elevated (>0.5)	1(0.5)	3(4.5)	4(1.6)	8.667	0.885	84.8	0.064	6.659	.510	86.8	0.148
TOTAL	188(100)	67(100)	255(100)								
**Total white Blood cells count (× 10^9^/L)**											
Normal (4-10)	102(54.3)	52(77.6)	154(60.4) Ref								
Low (<4)	81(43.1)	13(19.4)	94(36.9)	0.785	0.147	4.1	.776	0.450	0.035	5.842	.541
Elevated (>10)	5(2.7)	2(3)	7(2.7)	2.492	0.437	14.2	.304	1.862	0.115	30.22	.662
TOTAL	188(100)	67(100)	255(100)								
**Neutrophil absolute count (× 10^9^/L)**											
Low (<2)	66(35.1)	15(22.4)	81(31.8) Ref								
Normal (2–7)	111(59)	48(71.6)	159(62.4)	1.6	0.447	5.7	.470				
Elevated (>7)	11(5.9)	4(6)	15(5.9)	.841	0.255	2.7	.776				
TOTAL	188(100)	67(100)	255(100)								
**Lymphocyte absolute count (×l0^9^/L)**											
Low (<20)	87(45.3)	11(16.4)	98(38.4)								
Normal (20–40)	101(53.7)	56(83.6)	157(61.6) Ref								
TOTAL	188(100)	67(100)	225(100)								
**Basophil absolute count (× 10^9^/L)**											
Low (<0.01)	15(8)	8(11.9)	23(9) Ref								
Normal(0.01–0.1)	173(92)	59(88.1)	232(91)	0.639	0.258	1.5	.334	0.617	.208	1.825	.383
TOTAL	188(100)	67(100)	255(100)								
**Monocyte Absolute count (× 10^9^/L)**											
Low (<0.12)	21(11.2)	4(6)	25(9.8)	1.981	.654	5.9	0.227	1.129	.283	4.511	.864
Normal (0.12–1.2)	167(88.8)	63(94)	230(90.2) Ref								
TOTAL	188(100)	67(100)	255(100)								

COR=Crude Odd Ratio, OR=Adjusted Odd Ratio, CI=Confidence Interval

## DISCUSSION

The findings from this study suggest that patients with dengue have leucopoenia compared to non-dengue febrile patients. We have also demonstrated that lymphopenia is highly predictive of dengue positive results. This is similar to other studies conducted in other parts of the world.^15,24,25,26^ Elevated lymphocytes with atypical morphological features have been observed in some studies.^27,14^ However, we could not demonstrate the same in our study as we did not perform peripheral smears.

Elevated lymphocytes with atypical features have been associated with secondary dengue infection’ which indicates the role of immunological response’ and differences in cytokines produced during the first and subsequent disease exposure.^28,29^ This could be the result of augmented immune response due to secondary viral antigen exposure leading to a more severe form of the disease’a phenomenon also known as “the original antigenic sin”.^30^

Previous researches have demonstrated that cross reactive non neutralising antibodies from a previous dengue serotype bind to the new serotype and facilitate entry into the cells through Fc-receptors leading to activation of T-lymphocytes with subsequent lymphocytosis.^31,32^

Contrary to other studies, thrombocytopenia and elevated haematocrit were not significant in dengue positive patients compared to dengue negative patients. This could be due to the fact that these features are more common in patients with severe symptoms such and vascular leakage, haemorrhage and shock.^18,33^ This indicates that most of the patients in our study had a mild disease (classical dengue) with low likelihood of mortality.^33,28^

These findings (normal platelets, haematocrit and leucopoenia) with lymphopenia being predominant suggests that the current dengue infection could be the first exposure in most of the patients. Effective preventive measures are very important as further outbreaks with repeated infections will result into a more severe form of infection and possible increase in morbidity and case fatality.

## CONCLUSION

Leukopenia and lymphopenia are the most common findings in dengue patients in non-endemic areas like Dar es salaam. Lymphopenia is the most reliable predictor for dengue positive results among febrile patients with similar signs and symptoms. Case defining signs and symptoms combined with these haematological changes may be used by clinicians as a guide to order confirmatory test for patients who are suspected to have dengue infection.
